# High-Transmission Biomimetics Structural Surfaces Produced via Ultrafast Laser Manufacturing

**DOI:** 10.3390/biomimetics8080586

**Published:** 2023-12-04

**Authors:** Rui-Zhe Leng, Bi Yun, Zhi-Hao Chen, Chen Chai, Wei-Wei Xu, Yan-Hao Yu, Lei Wang

**Affiliations:** 1 State Key Laboratory of Integrated Optoelectronics, College of Electronic Science and Engineering, Jilin University, Changchun 130012, China; lengrz22@mails.jlu.edu.cn (R.-Z.L.); yunbi22@mails.jlu.edu.cn (B.Y.); czh_0920@163.com (Z.-H.C.); yanhao_yu@jlu.edu.cn (Y.-H.Y.); 2 GRINM Guojing Advanced Materials Co., Ltd., Langfang 065001, China; chaichen@guojing-tech.com; 3 School of Electrical and Information Engineering, Jilin Engineering Normal University, Changchun 130052, China; xuww@jlenu.edu.cn

**Keywords:** biomimetic structures, high transmission, antireflective surfaces, ultrafast laser manufacturing

## Abstract

Inspired by periodically aligned micro/nanostructures on biological surfaces, researchers have been fabricating biomimetic structures with superior performance. As a promising and versatile tool, an ultrafast laser combined with other forms of processing technology has been utilized to manufacture functional structures, e.g., the biomimetic subwavelength structures to restrain the surface Fresnel reflectance. In this review paper, we interpret the biomimetic mechanism of antireflective subwavelength structures (ARSSs) for high-transmission windows. Recent advances in the fabrication of ARSSs with an ultrafast laser are summarized and introduced. The limitations and challenges of laser processing technology are discussed, and the future prospects for advancement are outlined, too.

## 1. Introduction

Creatures have evolved many distinct properties in order to adapt to harsh living conditions by producing specific functional micro/nanostructures on the biological surfaces. For example, with hierarchical grating structures and fibers aligned on the surface, shark skin can reduce water resistance and biofouling [1,2,3]. Furthermore, due to abundant distributed hemispherical ommatidium on the compound eyes of insects, these exhibit excellent imaging abilities with a wide field of view, allowing insects to detect prey in motion [4,5]. Inspired by these structures, researchers have developed biomimetic functional surfaces, e.g., antireflective, superhydrophobic, wear-resistant and self-cleaning surfaces [6,7,8,9].

One important use of biomimetic subwavelength structures is to restrain surface Fresnel reflection for high transmission. As is well known, windows are necessary features in solar cells [10], photoelectric sensors [11], display screens [12] and so on. Among the properties of windows, their transmittance has one of the most important roles for optical equipment [13,14,15,16]. For instance, high transmission enhances the energy conversion efficiency of solar cells [17,18] and improves the performance of light-emitting diodes [19,20]. To optimize the optical characteristics, one solution is to deposit one or several coatings with certain thicknesses and refractive indexes on substrates [21]. By utilizing destructive interference between the reflected light from the upper and lower surfaces, coating technology shows outstanding antireflection performance aimed at a certain wavelength. However, these methods have limitations [22]. Although vapor deposition technology makes it possible to process large area surfaces, to achieve high transmittance in a certain waveband necessitates meticulous control over the thickness and refractive index of each individual layer, thereby adding complexity to the overall process. Additionally, inherent issues like thermal impedance mismatch scattering loss can result in peeling and underwhelming performance in terms of broad-waveband and wide-angle applications, thus further restricting its practical feasibility [23]. Therefore, an alternative approach is demanded to tackle the issues mentioned above.

Moth eyes, composed of thousands of subwavelength moth ommatidia aligned in an ingenious manner, have been discovered to effectively suppress reflectance and to have high transmission [24,25]. Inspired by this, researchers have been pursuing ways in which to replicate these antireflective subwavelength structures (ARSSs) on various materials for decades [26]. Substrates with ARSSs exhibit ultralow reflectance in a wide waveband as well as appreciable longevity [27,28]. Moreover, the flexibility of ARSS design is enhanced by various parameters, such as period, shape, fill factor and depth [29]. To date, many approaches have been proposed to prepare ARSSs, including photolithography [30,31], nanoimprinting [32], electron beam lithography [33,34], plasma etching [35,36,37,38], the sol–gel method [39], the self-assembly method [40] and laser processing [41,42,43,44]. However, there are still some limitations to be addressed, such as expensive equipment, complex manufacturing processes and inability to control the etching process. For example, chemical etching is easy to operate and inexpensive, but it is difficult to realize anisotropic etching. Meanwhile, photolithography combined with reactive ion etching exhibits high precision, but it cannot easily fabricate complex three-dimensional (3D) structures and can only process planar substrates [45]. Furthermore, the experimentation equipment and subsequent procedures required for the lithography method make it expensive and complex to implement.

As a mature processing method used for micro-optics and microfluidics [46], laser processing has the advantages of maskless, high-efficiency, non-contact and large-area processing regardless of material hardness, which is due to its ultrashort pulse duration and ultrahigh peak power density [47]. Furthermore, by adjusting the laser parameters of pulse number *N*, laser fluence *F*, polarization, etc., the optical performance of ARSSs determined by the period *Λ*, depth *d* and fill factor *f* can be adjusted in a wide range [48,49,50].

**Figure 1:**
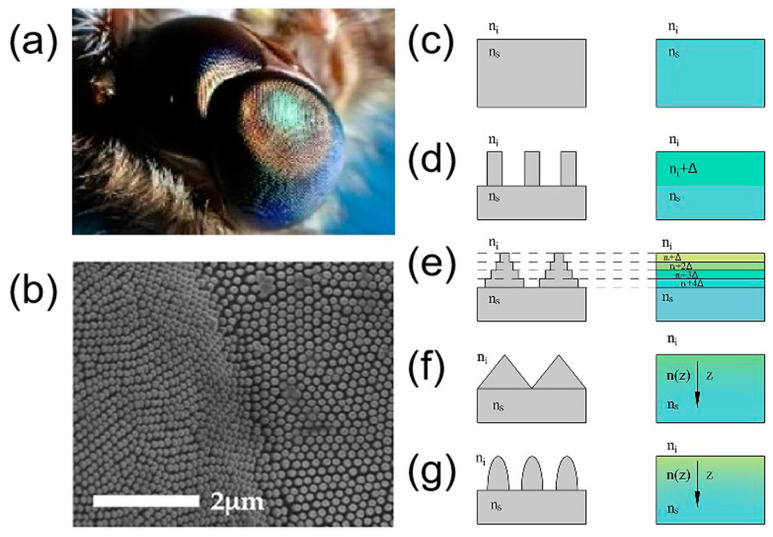
(**a**) Moth eyes; (**b**) scanning electron microscopy (SEM) image of the details of ommatidia; (**c**) flat substrate; (**d**) one-dimensional grating surface; (**e**) multilevel surface profile; (**f**) continuous conical surface profile; (**g**) continuous parabolic conical surface profile. (**a**,**b**) Reproduced from Sun et al. [65] and Müller et al. [48], with permission from Springer Nature and MDPI, respectively.

**Figure 2:**
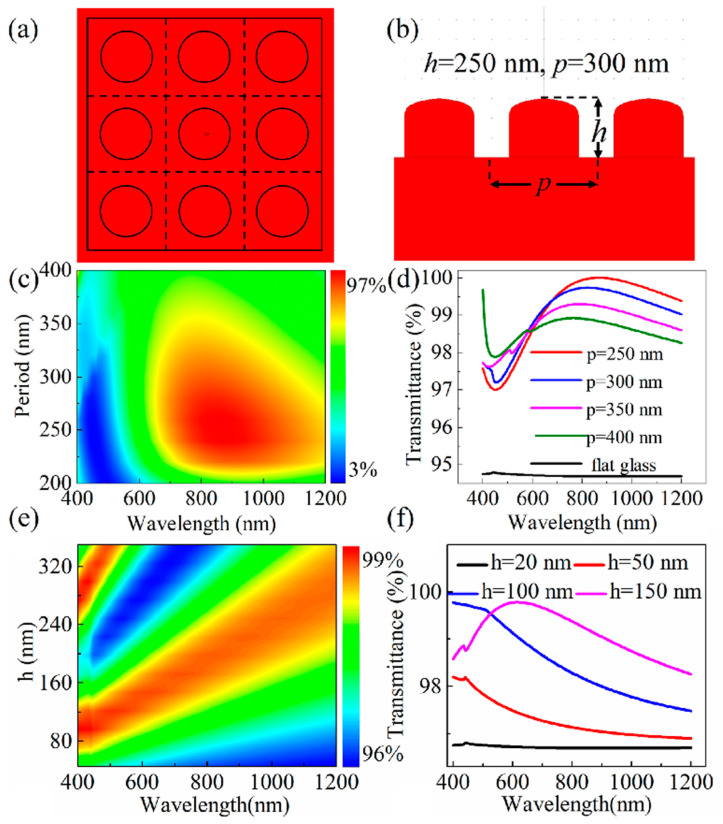
(**a**) Top view and (**b**) cross-section profile of the designed antireflective subwavelength structures (ARSSs); (**c**) Contour plot of transmittance as a function of period *p* and incident wavelength measured via rigorous coupled-wave analysis (RCWA); (**d**) corresponding simulated period-dependent transmittance spectra; (**e**) contour plot of transmittance as a function of height *h* and incident wavelength measured using RCWA; (**f**) corresponding simulated height-dependent transmittance spectra. Reprinted from Wang et al. [68], Copyright 2023, with permission from Elsevier.

**Figure 3:**
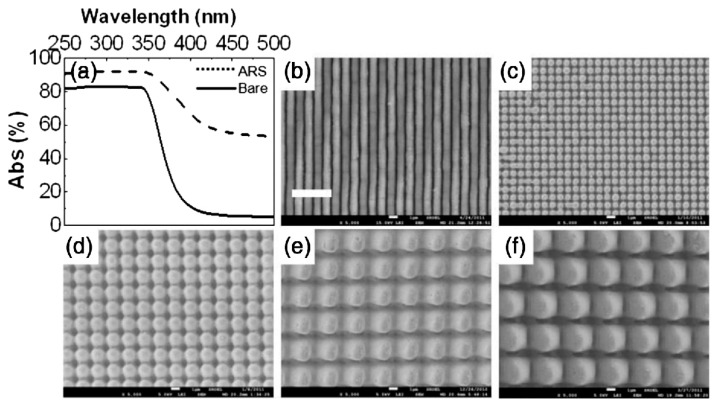
(**a**) absorption spectrum of zinc sulfide (ZnS) ranging from 200 to 500 nm; (**b**) gratings of period with 1μm; periodic square arrangement pillars of (**c**) 1 μm, (**d**) 2 μm, (**e**) 3 μm and (**f**) 4 μm. The scale bar for (**a**–**f**) represents 5 μm. Reprinted with permission from [72] © The Optical Society.

**Figure 4:**
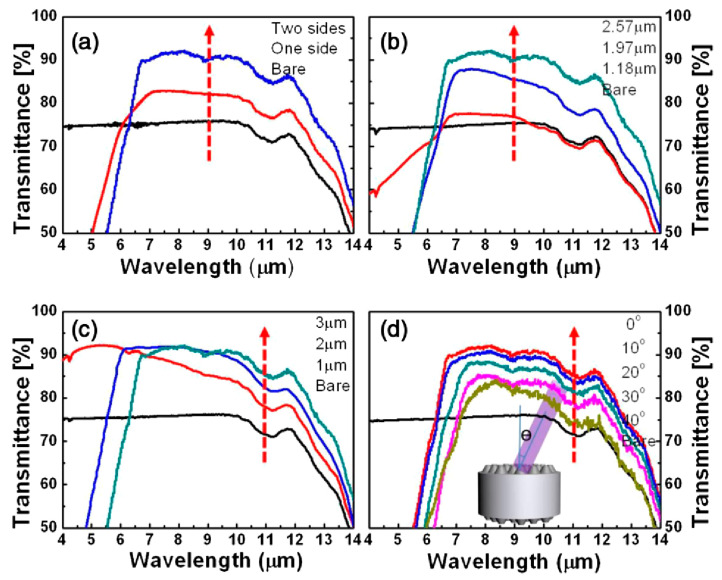
(**a**) Transmittance of single- and double-sided zinc sulfide (ZnS); (**b**) transmittance of double-sided ZnS with varying pillar depths; (**c**) transmittance of double-sided ZnS with varying periods; (**d**) transmittance of band double-sided ZnS at incident angles ranging from 0° to 40°. Reprinted with permission from [72] © The Optical Society.

**Figure 5:**
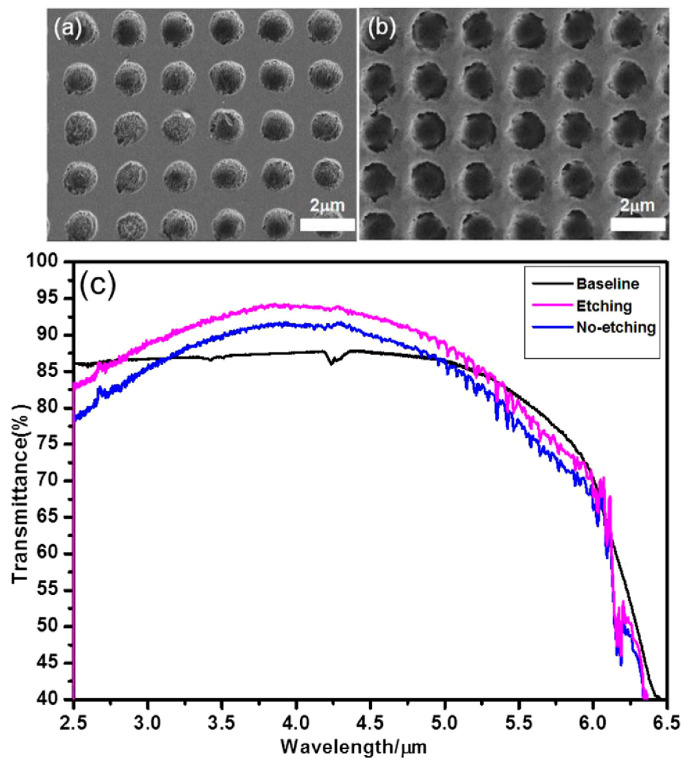
(**a**) Scanning electron microscopy (SEM) image of antireflective subwavelength structures (ARSSs) on sapphire without etching; (**b**) SEM image of ARSS on sapphire after etching; (**c**) measured transmittance of fabricated ARSS on sapphire with and without etching. Reprinted with permission from [77] © The Optical Society.

**Figure 6:**
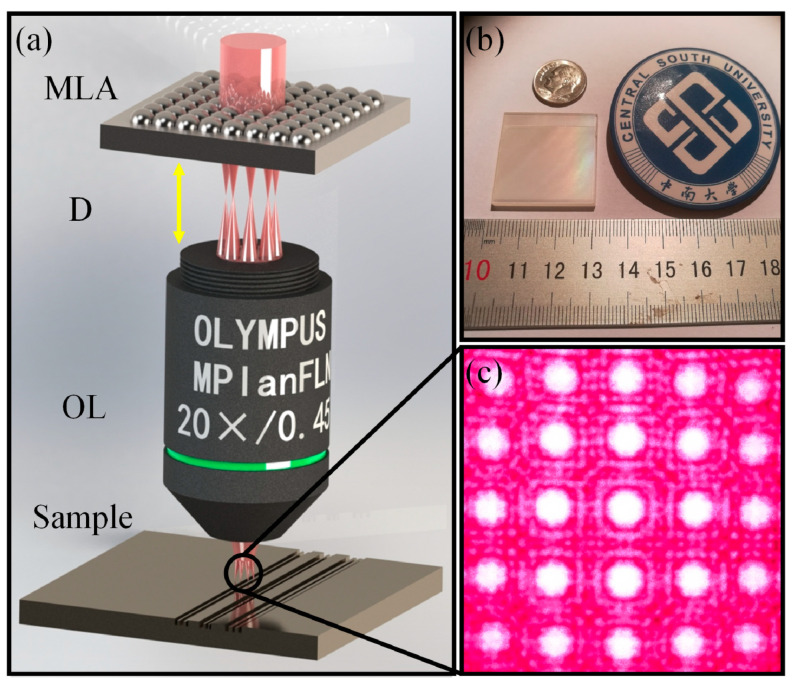
(**a**) The experimental setup for parallel femtosecond laser fabrication. MLA, microlens array; OL, objective lens; D, distance between MLA and OL; Sample, ZnS; (**b**) photograph of an ARSS sample fabricated using parallel femtosecond laser; (**c**) typical optical field intensity distribution of 5 × 5 foci diffraction pattern. Adapted from Zhang et al. [82], Copyright 2018, with permission from OSA Publishing.

**Figure 7:**
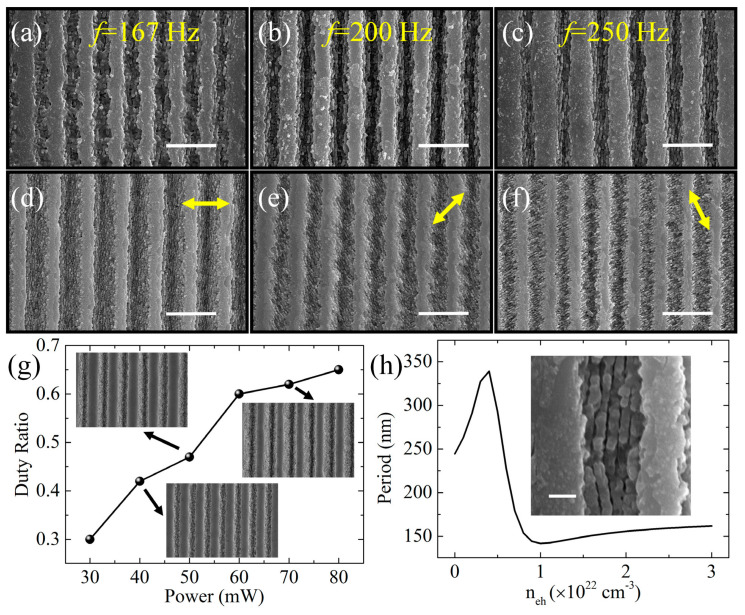
SEM images of microstructures were captured at different laser repetition rates: (**a**) 167 Hz, (**b**) 200 Hz, (**c**) 250 Hz. The morphologies of the microstructures were further analyzed with varying incident laser polarizations, as shown in (**d**,**f**). Laser polarization directions are indicated by yellow arrows; (**g**) duty ratio of the nanogratings’ area in the entire micrograting region under different laser energies. The inset is the SEM image of the fabricated surface corresponding to the laser energy; (**h**) simulated nanograting period versus excitation of electron. The inset is the SEM image of the typical nanograting. The scale bars in (**a**–**f**,**h**) represent 5 μm and 400 nm, respectively. Adapted from Zhang et al. [82], Copyright 2018, with permission from OSA Publishing.

**Figure 8:**
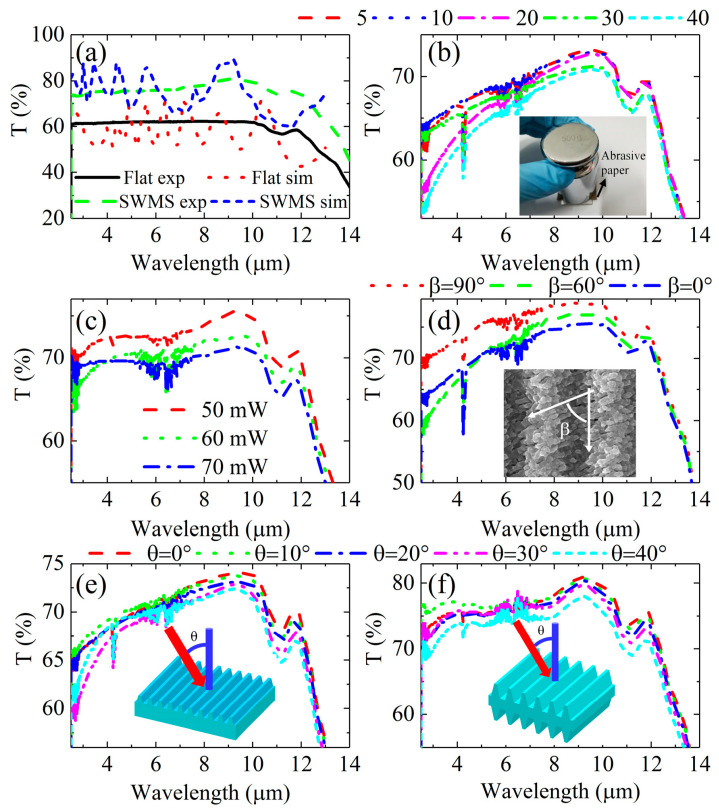
(**a**) Transmittance spectra of the flat zinc sulfide (ZnS) and antireflective subwavelength structures (ARSSs) were tested (exp) and simulated (sim); (**b**) transmittance spectra of the fabricated ARSS were examined after undergoing different circle-of-abrasion tests; (**c**) transmittance spectra of ARSS fabricated with different laser powers of 50 mW, 60 mW and 70 mW; (**d**) transmittance spectra of the ARSS were studied with different orientation angles β between the nanogratings and microgratings. The scanning electron microscopy (SEM) image inset displays the ARSS with an angle of β = 60°. The incident angle *θ*-dependent measured transmittance spectra of ZnS with a one-sided (**e**) or double-sided (**f**) ARSS. Adapted from Zhang et al. [82], Copyright 2018, with permission from OSA Publishing.

**Figure 9:**
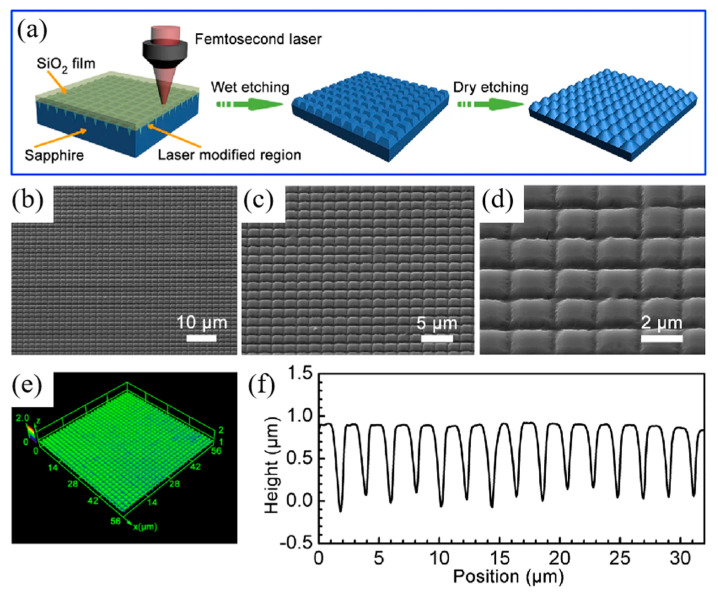
(**a**) Schematic illustration of the fabrication procedure of a sapphire surface with antireflective subwavelength structures (ARSSs); (**b**–**d**) SEM images of ARSS on sapphire; (**e**) three-dimensional (3D) morphology and (**f**) cross-section profile of the biomimetic sapphire surface with ARSS. Adapted from Liu et al. [89]. Copyright © 2022 Springer Nature.

**Figure 10:**
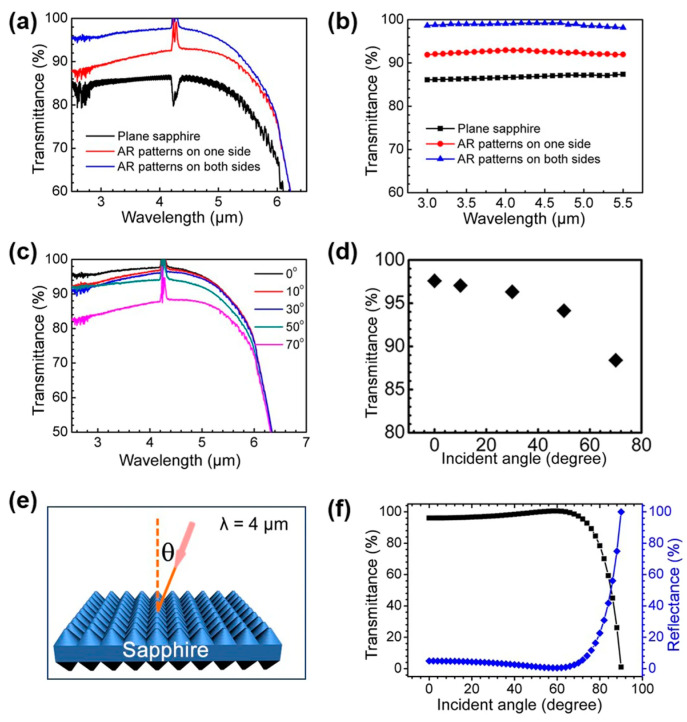
(**a**) Experimentally measured and (**b**) theoretically simulated transmittance of sapphire with antireflective subwavelength structures (ARSSs) on one side and both sides; (**c**) transmittance of the biomimetic sapphire surface with ARSS on both sides concerning incident angle; (**d**) relationship between transmittance of the ARSS and the incident angle at a fixed wavelength of 4 μm; (**e**) schematic illustration for the measurement and simulation of transmittance with different incident angles; (**f**) theoretically simulated transmittance and reflectance of the ARSS on both sides regarding the incident angle, with a fixed wavelength of 4 μm. Copyright © 2022 Springer Nature [89].

**Figure 11:**
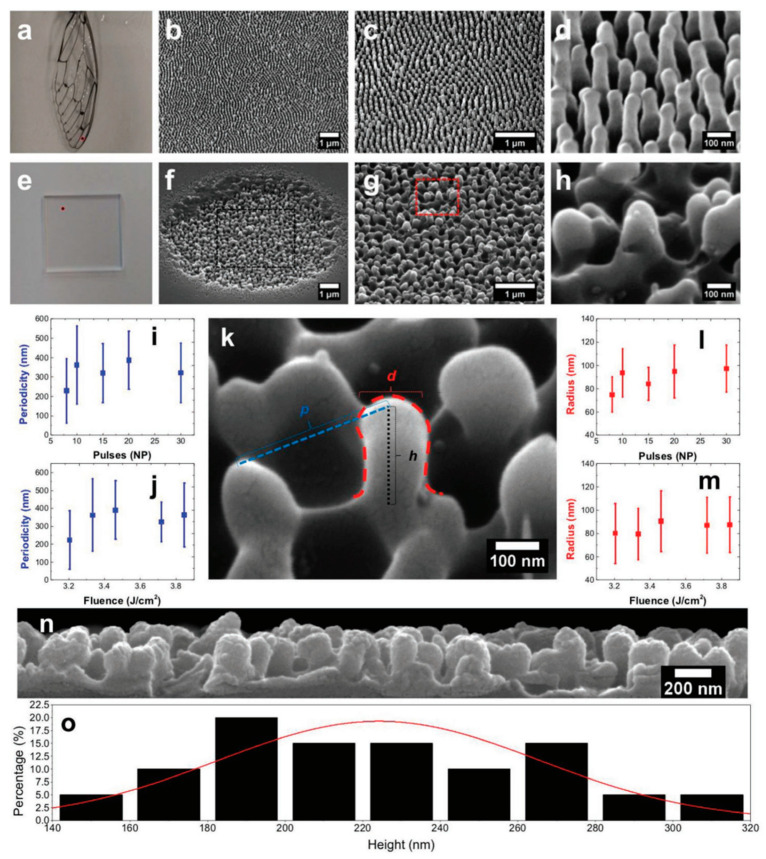
(**a**–**d**) Photograph of a Cicada Cretensis wing and scanning electron microscopy (SEM) images (45° tilted) at different magnifications showing the transparent antireflective area, with the red spot indicating the SEM imaging area; (**e**–**h**) photograph of a fused silica plate and SEM images (45° tilted) showing a spot fabricated on the surface, with the red spot indicating the location of irradiation; (**i**) period of nanospikes as a function of pulses number of NP; (**j**) period of nanospikes as a function of fluence at number of pulses NP = 10; (**k**) SEM images (45° tilted) of a single nanospike; (**l**) radius of nanospikes as a function of NP for fixed fluence (FI) = 6.6 J/cm^2^; (**m**) radius of nanospikes as a function of fluence for NP = 10; (**n**) cross-section SEM image of the femtosecond-laser-induced nanospikes; (**o**) height distribution. Reprinted from Antonis et al. [95], Copyright 2019, with permission from Wiley.

**Figure 12:**
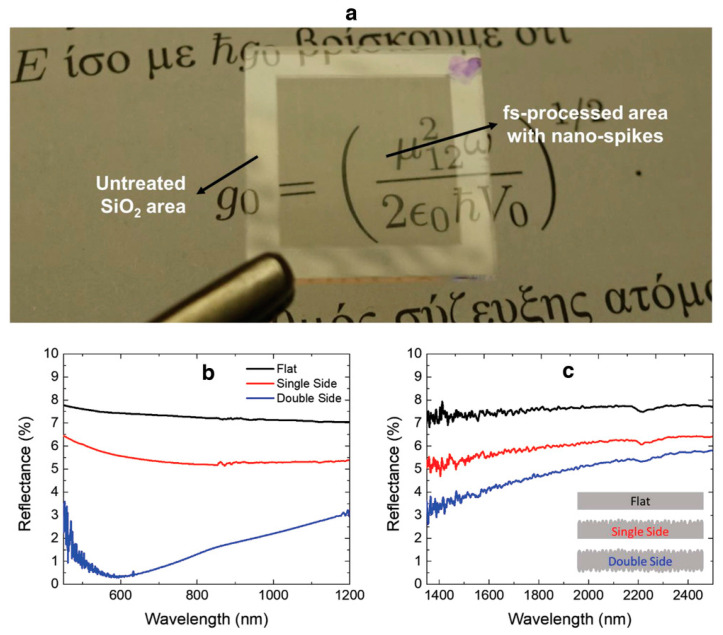
Antireflection measurement (**a**) A photograph of a fused silica sample plate, with the central part being subjected to laser treatment to create nanospikes. The reflectance of the flat and laser-treated areas on one or both sides of the fused silica plate is shown in (**b**,**c**). Reprinted from Antonis et al. [42], Copyright 2019, with permission from Wiley.

**Figure 13:**
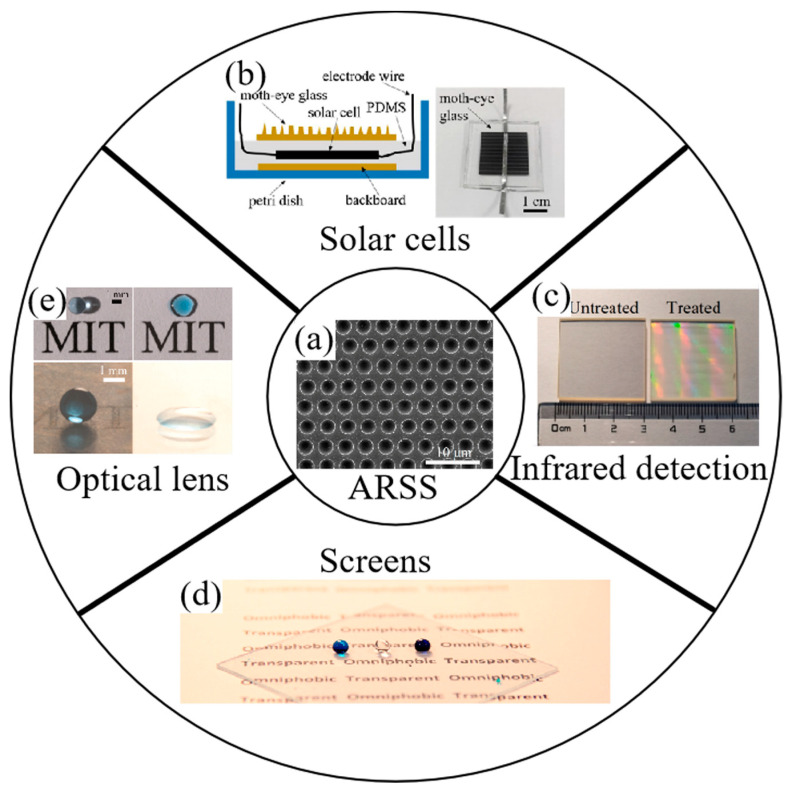
Applications of ARSS in some fields. (**a**) ARSS produced by femtosecond laser on ZnS. (**b**) Photography of the encapsulated PV module. (**c**) Photography of flat ZnS and laser-treat ZnS for infrared detection. (**d**) Transparent antireflection surface with high contact angle, with images of three liquid droplets: water (bottom middle), oleic acid (bottom left), and hexadecane (bottom right). (**e**) Water droplets on a transparent surface with ARSS and on flat glass. The very low reflectance and high water contact angle of the surface with ARSS contrast intense reflection and low water contact angle on flat glass. (**a**) Adapted from Wang et al. [71], with permission from MDPI; (**b**) adapted from Luo et al. [100], with permission from Elsevier; (**c**) adapted from Wang et al. [70], with permission from Elsevier; (**d**) adapted from Mazumder et al. [101], with permission from RightsLink; (**e**) adapted from Kyoo-Chul et al. [13], with permission from Rightslink. All rights reserved.

Herein, we provide a concise introduction to the antireflective mechanism of biomimetic subwavelength structures (Figure 1 and Figure 2). We collect the recent advancements and innovative techniques in the fabrication of ARSSs using ultrafast laser manufacturing technology (Figure 3, Figure 4, Figure 5, Figure 6, Figure 7, Figure 8, Figure 9, Figure 10, Figure 11 and Figure 12). At first, early work concerning nanosecond laser interference ablation on zinc sulfide (ZnS) is introduced (Figure 3 and Figure 4). Then, ultrafast femtosecond laser processing on sapphire is presented, as well as a comparison between nanosecond laser processing and ultrafast femtosecond laser processing (Figure 5). Next, solutions to low processing efficiency, low surface quality and difficulties in fabrication of high-aspect-ratio structures are presented, as well as the antireflective nanostructures for visible waveband (Figure 6, Figure 7, Figure 8, Figure 9, Figure 10, Figure 11 and Figure 12). Concerning the applications of ARSSs in daily life, such as for optical imaging and displays, enhancement of the performance of photovoltaic modules, and infrared detection, corresponding recent work and progress are mentioned (Figure 13). Finally, the challenges and the prospects of the laser processing technology for ARSSs are discussed.

## 2. Mechanism of Antireflection for High-Transmission Structural Surfaces

Fresnel reflection is a general phenomenon taking place at the interface between two different media (such as air and glass) when a light beam irradiates. In particular, there are two ways to reduce reflectance: increase the absorbance by using hybrid structures (structures with scales ranging from subwavelength to tens times of wavelength) [51] or increase the transmittance by using ARSSs [52]. The former option takes effect as follows when the light confronts the hybrid structures. Light will reflect multiple times inside the structures at the scale of ten times the wavelength, while the nanostructures will enhance the absorption coefficient [53,54,55]. In this way, the absorbance can be enhanced significantly via optical trapping, resonance and effective surface extension [56,57,58]. Efforts to enhance absorbance are applied to semiconductor/metallic materials, where the reflectance is the essential key parameter, e.g., black silicon, colorful metals [59,60,61,62,63]. The latter option, which we mainly discuss in this paper, is applied to transparent materials to improve the transmittance by changing the effective refractive index. It is believed this reflection is caused by the refractive index difference or the deeper physics from the different electron densities and intrinsic electron oscillation frequencies of different materials [64]. The reflectance *R* or transmittance *T* can be computed by applying the Fresnel equation, with the underlying assumption that the larger the change in refractive index Δ*n*, the greater the reflection *R* [52]. In practical applications, the reflected light will cause substantial wastage of energy as well as send a ghost image to systems. Therefore, minimizing the reflected and scattered light at the material interface is highly desirable.

To date, methods to diminish the reflected light can be classified into two categories: layer coatings and antireflection structures (Figure 1a,b). To coat layers is a simple way to reduce the reflectance *R* by introducing one or more thin films. Each layer is expected to have a designed refractive index and thickness. According to the Fresnel equation, the reflection coefficient *r* for a single film is [66]

(1) $$r=\frac{n_{1}\left(n_{i}-n_{s}\right)cosk_{0}h+i\left(n_{i}n_{s}-n_{1}^{2}\right)sink_{0}h}{n_{1}\left(n_{i}+n_{s}\right)cosk_{0}h+i\left(n_{i}n_{s}+n_{1}^{2}\right)sink_{0}h}$$

where $\theta_{i}$, $\theta_{r}$ and $\theta_{t}$ are the incident, reflected and refractive angle, respectively; $n_{i},\;n_{s}\;and\;n_{1}$ are the refractive indices of air, substrate and film, respectively; *k*_0_ is the propagation constant; *h* is the thickness of film; and $i=\sqrt{-1}$. From Equation (1), we can derive the reflectance *R* [66]

(2) $$R=\frac{n_{1}^{2}{\left(n_{i}-n_{s}\right)}^{2}{cos}^{2}k_{0}h+{\left(n_{i}n_{s}-n_{1}^{2}\right)}^{2}{sin}^{2}k_{0}h}{n_{1}^{2}{\left(n_{i}+n_{s}\right)}^{2}{cos}^{2}k_{0}h+{\left(n_{i}n_{s}+n_{1}^{2}\right)}^{2}{sin}^{2}k_{0}h}$$

We can produce a concise expression when $k_{0}h=\pi /2$, where the optical phase of the film is (2m-1)*λ*_0_/4 at m = 1, 2, 3…. In the case of *h* = *λ*_0_/(4*n*_1_), we can ascertain that [52]

(3) $$R=\frac{{\left(n_{i}n_{s}-n_{1}^{2}\right)}^{2}}{{\left(n_{i}n_{s}+n_{1}^{2}\right)}^{2}}$$

which equals zero at [52]

(4) $$n_{1}^{2}=n_{i}n_{s}\;\mathrm{or}\;n_{1}=\sqrt{n_{i}n_{s}}$$

One can calculate the minimum height from Equation (2), and *h* is chosen as *λ*_0_/(4*n*_1_) [52]. Therefore, to obtain the minimum reflectance, the thickness and the refractive index of the film should be controlled accurately. Cryolite (*n* = 1.35) and magnesium fluoride (*n* = 1.38) are general low-index films. MgF_2_ (*n* = 1.3–1.4) is used more frequently since it is more durable. As for the typical substrate of glass (*n* ≈ 1.5), the refractive indices of the films above are still too large to satisfy Equation (4), even though a single *λ*_0_/4 layer of MgF_2_ can reduce the reflectance of glass from about 4% to a bit more than 1%.

Physically, the ARSS can be considered as a subset of successive and infinite layers that have a gradient refractive index from the air to the substrate, as shown in Figure 1e. Figure 1d shows the schematic of subwavelength gratings equivalent a new layer with a gradient refractive index of *n_i_* + Δ*n*. Under this circumstance, the Fresnel refraction is restrained and high transmission is achieved [67]. According to effective medium theory (EMT) [52], the effective refractive index can be described in terms of a mixture of two materials’ physical constants (permittivity ϵ, permeability μ and conductivity σ) between the air and subwavelength structures. In other words, the transmission of ARSS is determined by the effective refractive index along the Z-direction, which is determined by the weighted average material in one period of subwavelength structures (filling factor) at a certain height. For the case of a multilevel surface profile, the structures are taken as a film stack, each of which corresponds to a distinct level with a continuously changing filling factor in depth (Figure 1e). For a continuous profile (Figure 1f,g), when considering the ARSS as infinite layers of film, the effective refractive indices $n_{eff}$ of each can be expressed by [52]:

(5) $$n_{eff}=\sqrt{n_{s}^{2}f+n_{i}^{2}(1-f)}$$

where *f* is the filling factor (area fraction) of the material in each period. We can include that the curve of the effective refractive indices increases with the vertical distance. By designing the structures properly, we can ensure that the gradient index can be sufficiently smooth, so that ∆*n* between adjacent films approaches zero, meaning reflection can be eliminated dramatically.

As a particular grating, the ARSS is designed to only propagate, reflect or refract the 0th order of light. According to the EMT, at a given wavelength and for incident angle $\theta_{i}$, a ceiling of the period-to-wavelength ratio *Λ*/*λ* can be expressed by [67]

(6) $$\frac{\Lambda }{\lambda }<\frac{1}{\max\left[n_{s},n_{i}\right]+n_{i}sin\theta_{max}}$$

where *Λ* is the period of the ARSS, and $\theta_{max}$ = 90° is the maximum incident angle. From Equation (6), the period of the ARSS for a typical wavelength can be properly designed. The period of the ARSS determines the range of the operating wavelength, while the depth and shape of the ARSS dictate the antireflection performance. Additionally, a film depth exhibits zero reflection at the normal incidence only if the depth is higher than [67]:

(7) $$d_{min}=\frac{\lambda }{4\sqrt{n_{i}n_{s}}}$$

In summary, the performance of the ARSS is influenced by parameters such as period, fill factor, depth and contour shape. The period *Λ* determines the propagation direction of diffraction orders. When the period is larger than *λ*⁄(*n_s_* + *n_i_*), the high-order diffraction effects will affect the transmission of optical energy. The fill factor influences the effective refractive index of the ARSS, normally resulting in a shift in the transmission spectrum. A too low or high fill factor will make it difficult to achieve an appropriate effective refractive index. In addition, the fill factor also affects the structure’s depth in practical manufacturing. For designs with a small fill factor, a greater depth can be achieved, while a large fill factor reduces the depth achievable through etching technology. Changes in structure depth directly affect the longitudinal refractive index gradient distribution of the ARSS, and the depth parameter is directly related to the antireflective performance. The effect of each parameter on transmittance can be studied through numerical calculation methods, and comprehensive structural parameters can be designed based on trends [68].

By means of rigorous coupled-wave analysis (RCAW), optimal parameters of the ARSS can be attained. In 2023, Wang et al. analyzed the impact of structural parameters on transmittance from the visible to near-infrared bands [68]. For wavelengths ranging from 400 nm to 1200 nm, the initial height is set as 300 nm to explore the periods of the reflectance. Figure 2a–d show the calculated transmittance for the ARSS of different periods. The trend of transmittance changing with the structural period in the visible light range is consistent with the EMT. The observed red-shifted tendencies in Figure 2d provide an approach for antireflective band sectioning by modulating the periods. Similarly, Figure 2e,f display the relationship of the ARSS with heights from 40 nm to 360 nm at a period of 300 nm. The transmittances at the wavelength bands from 600 nm to 1200 nm are enlarged with the structure height increasing. This phenomenon can be interpreted on the basis that the formation of a smooth refractive index gradient on the surface can effectively suppress the Fresnel reflection.

Therefore, it is crucial to satisfy the requirement of the depth and period of the ARSS. To date, various ultrafast laser manufacturing technologies have been utilized to fabricate ARSSs on window materials, e.g., silica, sapphire, diamond, zinc sulfide and so on.

## 3. Ultrafast Laser Fabrication of ARSS

Zinc sulfide (ZnS) is a kind of prominent far-infrared transparent material, mainly used for infrared detection and guidance [69,70]. However, due to its high refractive index (*n* = 2.2 at *λ* = 9 μm), a large portion of the incident light is reflected, resulting in low infrared imaging quality. The ARSS provides a feasible route to solve the problem [71]. In 2011, Wang et al. proposed a simple approach for rapid and maskless fabrication of ARSSs with high transmittance and broadband transmittance [72]. Their approach involved using multiple exposures of a two-beam interference ablation (TBIA) technique to create planar structure patterns with a high aspect ratio. In their experiment, a laser with an emission wavelength of 355 nm in the material absorption waveband (Figure 3a), a frequency of 10 Hz and a pulse duration of 10 ns was used. In the first step, one-dimensional gratings were obtained through TBIA. In the subsequent step, the substrate was rotated at a certain angle along the normal surface to fabricate the desired patterns. The period of the grating could be adjusted by changing the angle between the two laser beams, *Φ*, as per the desired design.

(8) $$\Lambda =\frac{\lambda_{F}}{2\sin\left(\Phi /2\right)}$$

where *λ_F_* is the laser wavelength. By changing the rotation angle and the exposure pulses, the gratings with different periods were obtained. After double exposure of TBIA, they obtained arrays of micropillars with different periods of *Λ* = 1 μm (Figure 3b,c), *Λ* = 2 μm (Figure 3d), 3 μm (Figure 3e) and 4μm (Figure 3f), which resulted from *Φ* = 10.18°, 6.78° and 5.09°, respectively. According to Equation (7), given *n*_s_ = 2.2 for ZnS at a typical infrared wavelength of *λ* = 10 μm, we have *h* = 1.67 μm. The heights of ARSSs achieved in their experiment reached 2.57 μm, which is far more than the height designed by Equation (7). To minimize the reflection to the most extent, a double-sided structured surface was fabricated. The transmittance measured using Fourier transform infrared spectrometry is shown in Figure 4. The results indicate that with structures of *Λ* = 1, 2 and 3 μm, the wavebands of transmittance exceeding 90% are located at 4.32–6.85 μm, 6.99–8.84 μm and 8.92–10.15 μm. Even for *θ* = 40°, the bandwidth of transmittance over 80% is from 7.4 to 10.2 μm, which is difficult to achieve by utilizing multilayer coating technology.

Since the pulse duration of a short pulsed laser is quite narrow, the peak intensity is extremely high, enabling the removal of materials through localized heating (nanosecond pulses or some long pulsed ultrafast picosecond pulses) or the plasma effect (ultrafast femtosecond pulses) [73,74,75]. Normally, due to the thermal diffusion effect of nanosecond laser manufacturing, the feature size resolution is limited to the microscale. Attributed to the multiphoton absorption effect, a femtosecond laser, which has at least six times the power of a nanosecond laser in peak power intensity, enables the diffraction limitation to be removed for the demonstration of nanoscale structures [36]. In this way, a ultrafast femtosecond laser rather than a nanosecond laser is a promising option to process materials with high thermal stability such as sapphire and diamond [76]. Over the past decade, ultrafast femtosecond lasers based on multiphoton absorption have been proven to offer a robust processing method for three-dimensional (3D) micro/nanostructures of almost any material [74]. Generally, ultrafast laser direct-writing technology can be divided into two types: additive manufacturing and subtractive manufacturing [45]. The former method is commonly used for processing polymer materials, while the latter method is mainly used for material removal using a tightly focused objective lens [45]. By selectively modifying the target region through laser irradiation, a difference in etching rate can be induced between the laser-modified region and the original region. In the following, we will introduce some classic work on high-transmission ARSSs utilizing ultrafast laser manufacturing.

In 2017, Li et al. proposed a simple approach for the production of ARSSs on sapphire with high transmittance and broadband transmittance in the mid-IR. Their approach involved femtosecond laser direct-writing assisted with wet etching [77]. In their experiment, they used an objective lens with certain specifications to tightly focus the femtosecond laser on a sapphire sample of a specific thickness. Laser pulses at a wavelength of 400 nm were utilized for fabrication. Given *n*_s_ = 1.77 for sapphire at a typical infrared wavelength of 5 μm, the minimum depth of the ARSS determined using Equation (7) is 0.71 μm. They successfully produced inverted pyramid and cone arrays with a pitch of 2 μm and height of 900 nm on sapphire. To further optimize the surface roughness, one sample was etched for 6 min in a mixture of sulfuric and phosphoric acid (3:1), and then compared with another without etching. The morphologies of the two samples are shown in Figure 5a,b. After etching, the debris on the surface was removed effectively, diminishing the diffuse reflection. The measured transmittance is shown in Figure 5c, and a bare substrate was measured as a reference, too. It can be observed that there is an increase in the total transmittance after etching over a specific spectral range. At a specific wavelength, the transmittance reaches a maximum value of 92.5%. In 2020, Liu et al. reported a novel laser processing strategy to realize high-aspect-ratio crack-free microstructures on sapphire [78]. This strategy combines femtosecond-laser-induced plasma-assisted ablation and subsequent laser ablation. The researchers achieved crack-free and taper-free microgrooves on sapphire with a maximum aspect ratio up to 10:1.

However, as a point-by-point direct-writing technology, the fabrication efficiency of the femtosecond laser is currently limited, which hinders its practical applications [45]. To address this issue, various strategies have been proposed to improve the fabrication efficiency. Recent studies utilize parallel processing technology, including laser interference [79], line light field processing [80] and multi-focal-point processing [81]. In a study conducted by Zou et al. in 2020, a high-speed, large-area and uniform fabrication method for micro/nanograting structures on graphene oxide film was demonstrated [80]. By employing cylindrical focusing of a femtosecond laser on graphene oxide film, they were able to produce uniform subwavelength grating structures at a high speed, while simultaneously conducting an in situ photoreduction process.

Another approach was proposed by Zhang et al. in 2018, which utilized a femtosecond laser parallel multibeam to achieve the desired and optimal structures [82]. With the assistance of a microlens array (MLA), the incident single-foci light field was converted into a multi-foci pattern on the surfaces. In their experiment, a femtosecond laser with a wavelength of 800 nm, pulse duration of 120 fs and a tunable frequency ranging from 1 to 1000 Hz was employed. Prior to focusing with an objective lens (NA = 0.45, 20×), the incident beam was spatially split by the MLA (period = 150 μm and focal length = 5.3 mm), as shown in Figure 6a. An intensity distribution of a 5 × 5 foci array captured by a CCD camera is shown in Figure 6c. In this method, a typical 28 mm × 28 mm structure with rainbow colors on ZnS can be completely scanned within 7 h, as shown in Figure 6b. The influence of processing parameters such as the distance between the objective lens and MLA, frequency, polarization and laser power on the morphology of ARSS was also studied. The pulse energy and scanning speed were fixed at 45 μJ and 1 mm/s, respectively. By implementing these advancements, it is expected that the fabrication efficiency of a femtosecond laser can be significantly improved, thereby expanding its practical applications.

Figure 7a–c reveal the evolution of morphology with different laser repetition frequencies. When the repetition frequency is 167 Hz, there is no polarization-dependent nanograting, as shown in Figure 7a. With increasing repetition frequency, nanogratings appear and spread in the laser ablation trace, as shown in Figure 7b,c. The orientation of nanogratings can be freely adjusted by changing the laser polarization, as depicted in Figure 7e,f. Laser polarization is indicated by yellow arrows, and it can be observed that the nanograting orientation is always perpendicular to the laser polarization. The duty ratio of the nanogratings relative to the microgratings varies along with the laser power, as shown in Figure 7g. At a fixed scanning speed of 1 mm/s, the duty ratio increases from 0.30 to 0.66 as the laser power increases from 30 mW to 80 mW. The relationship between the period of the nanogratings and the excited electron density *n_eh_* is illustrated in Figure 7h.

The transmittance spectra measured are presented in Figure 8. Compared to flat ZnS, the transmittance of ARSS increases in the wavelength range of 3–12 μm. Figure 8e,f show the angle-dependent transmittance spectra of one-sided and double-sided ARSSs with a period of 3.2 μm and a depth of 1 μm. The results demonstrate that the transmittance of double-sided ARSSs is approximately 76.5% in the range of 4–10 μm, which is higher than that of one-sided ARSSs (approximately 71%). Even at an incidence angle of up to 40°, the transmittance of both samples remains above 72.2% and 77.7% at a wavelength of 9 μm, respectively. For a one-dimensional grating profile, the duty cycle is identical to the filling factor. By changing the duty cycle, the effective refractive index changes. Once the effective refractive index and depth satisfy Equations (4) and (7), respectively, minimum reflection will be achieved. In Zhang’s experiment, the performance of ARSSs is limited by the depth of gratings.

Under femtosecond laser irradiation, a large number of deposited particles and pieces of debris are typically generated on the substrate surface around the ablation area, and nanogratings will be induced inside the structure. These defects can cause light to be reflected and scattered, negatively affecting the morphology and roughness of the structures [83]. In particular, the light scattering caused by nanogratings significantly limits the fabrication of high-aspect-ratio structures [84]. Most of the current reports on ARSS processing with femtosecond lasers are conducted in ambient or vacuum atmospheres. However, these special environments result in complex procedures and expensive costs, limiting the application of femtosecond laser direct writing. To address this challenge, several methods have been proposed [85,86,87,88]. In 2020, Chen et al. reported an economical and simple method for fabricating multiscale micro–nano composite structures by using laser-cleaning-assisted femtosecond laser ablation on silicon surfaces in ambient air [83]. In their experiment, a cylindrical lens (*f’* = 75 mm) was used to obtain an elliptical laser spot with a long axis size of 4mm and a short axis size of 8.28 μm. The continuous decay of energy during multiple laser cleanings, oxide deposition and laser redeposited particles were removed effectively. Finally, the average reflectance in the wavelength range of 300–2500 nm was reduced to 2.06%, achieving a reflection below 5.0% from 2.5 to 10 μm.

By means of etching technology, particles and debris can be removed effectively. Recent studies have shown that combining femtosecond laser modification with subsequent etching procedures is an effective approach for precisely controlling the surface morphology of ultrahard materials [45,46,90,91]. In 2022, Liu et al. [89] introduced an inside-out femtosecond laser deep-scribing technology for manufacturing biomimetic sapphire windows with microstructures on the surface. To prevent particle and fragment generation during the laser scanning process, a silicon oxide (SiO_2_) sacrificial layer was used. The SiO_2_ sacrificial layer and laser-modified region can be removed using HF aqueous solution, resulting in high-quality antireflective surface structures (ARSSs). Dry etching processes were carried out using an inductively coupled plasma system, as shown in Figure 9a. Scanning electron microscope images showed the uniform distribution of closely packed ARSSs over a large area, as shown in Figure 9b–d. According to Equations (6) and (7), the period of ARSSs should be less than 2.26 μm for a typical infrared wavelength of 4 μm, and the height of ARSSs should be larger than 0.75 μm. In their experiment, the height of the ARSSs was approximately 1 μm (Figure 9f), which is more than the optimal height calculated with Equation (7). Compared to flat sapphire windows, sapphire windows with ARSSs exhibited significantly increased transmittance in the mid-infrared range from 2.5 to 6 μm (Figure 10a). The transmittances of sapphire with an ARSS on both sides or one side were 98% and 92% at 4 μm, respectively, both higher than that of the flat sapphire window. The transmittance of sapphire with ARSSs slightly decreased with an increasing incidence angle up to 50°, but was still maintained at over 95% at 4 μm. However, increasing the incidence angle to 70° resulted in a significant decrease in transmittance to 89% at 4 μm (Figure 10c,d). Theoretically, this sacrificial layer can overcome huge problems faced when using direct ablation and demonstrate finer ARSSs if spatial light modulation is involved.

The application of ARSSs in the visible light range is of great significance, but it is still challenging to demonstrate ARSS through ultrafast femtosecond laser manufacturing. According to EMT, the period of ARSSs for visible light is limited in the range of 150–250 nm. Although the laser can break the optical diffraction, it is hard to fabricate ARSSs with specific parameters at the nanoscale arbitrarily and accurately, e.g., controllable cutting kerf and variable spatial ratio and height. Furthermore, the feature size resolution of ARSSs is limited to the microscale. 

In 2020, Li et al. (2020) proposed an optical far-field-induced near-field breakdown (O-FIB) method as an optical alternative to the conventional focused ion beam (FIB) technique [84]. This technique utilizes polarization control to steer nanogroove writing along the desired pattern, and a spatial resolution of less than 20 nm is readily achieved. This approach provides an entirely new concept in the production of nanopatterns using femtosecond laser direct writing. The combination of the femtosecond laser field and material properties enables the production of features with sizes at the nanoscale. Laser-induced periodic surface structures (LIPSSs) has been proven to be versatile tools for the fabrication of nanostructures [92,93,94].

In 2014, Long et al. used a picosecond laser to fabricate large-area LIPSSs on copper surfaces [96]. With suitable combinations of laser parameters, LIPSSs with different morphologies were fabricated and different structural colors. Because of the existence of abundant nanostructures, LIPSSs showed an enhanced super-hydrophobicity and reduced adhesive force to water, with a large contact angle (153.9 ±  3.2°) and sliding angle (11 ±  3°). In 2019, Antonis et al. proposed a single-step approach for the realization of omnidirectional transparent antireflective glass [95]. Figure 11a–d show the photography of a wing, and SEM images show the transparent antireflective area at different magnifications. A parametric study was conducted to determine the appropriate parameters required for the nanopillars’ formation. With a fixed fluence (FI), different circularly polarized numbers of pulses (NPs) are required for well-ordered nanospikes. Figure 11e–h show the photography of a fused silica plate and SEM images at different magnifications, where the red spot indicates the location of irradiation. A morphological analysis was conducted to account for the period, radius and height of the fabricated nanospikes (Figure 11k). The above results are listed in Figure 11i–o. According to the analysis of the corresponding SEM images, the period was estimated to be within in the range of 200–400 nm (Figure 11i,j), the radius was measured in the range of 70–100 nm (Figure 11l,m) and the average height was estimated to be 224 ± 41 nm (Figure 11n,o). Figure 12a presents a picture of a square-shaped area of nanospikes processed in the central part of a silica plate. The laser-treated area exhibits a significantly higher level of antireflection compared to the bare area. Moreover, treating both sides of the silica plate further reduces the reflectance. The difference in reflectance between the untreated and single-side-treated areas is 1.7% for 1200 nm and 1.9% for 600 nm, respectively. In the case of the double-side-treated area, the difference in reflectance is 4.1% for 1200 nm and 7.1% for 600 nm (Figure 12b). For wavelengths in the range of 400–800 nm, the ARSS shows a broadband reflection reduction. In the near-infrared spectrum, laser-treated area shows higher transmittance compared to the bare flat (Figure 12c). This effect can be attributed to the nanospikes’ randomness. Moreover, the antireflective behavior can be attributed to a transition of the effective refractive index induced by nanospikes. This single-step and chemical-free technique allows for the creation of artificial structures with impressive antireflective properties in the visible and near-infrared wavelength range. Table 1 lists the fabrication, advantages and optical properties of ARSSs on different materials. Of particular note, by using inside-out femtosecond laser deep-scribing technology, Liu et al. provided a concept for how to effectively avoid uncontrollable surface damage and maintain undamaged regions. Furthermore, by utilizing LIPSSs, Antonis et al. achieved fabricating ARSSs at the nanoscale, realizing an antireflective effect in the visible range.

**Table 1:** Summary of ARSSs fabricated using ultrafast lasers.

Materials	ARSS	Methods	Laser Width	Advantages	*T*_d_; *E*_ff_	Waveband (μm)
ZnS [72]	Tall sinusoidal pillars	A multiple exposure of the two-beam interference	10 ns	Maskless, rapid and simple; one-step approach for ARSS	>90% 20%	4.32–6.85
Sapphire [77]	Inverted cone and pyramid arrays	Femtosecond laser direct writing assisted with wet etching	120 fs	An inexpensive, maskless and reproducible way to fabricate an ARSS on sapphire	95% 9%	3–5
ZnS [82]	Nanogratings embedded in microgratings	Femtosecond laser parallel multibeam ablation	120 fs	Improved efficiency through the utilization of laser energy in multibeam energy	>76.5% 12%	4–10
Sapphire [89]	Pyramidal structure array	Inside-out femtosecond laser deep-scribing technology	290 fs	Effectively avoids uncontrollable damage; high-quality ARSS with an aspect ratio of 80	95% 10%	2.5–6
Fused silica [95]	Arrays of nanopillars	Laser-induced periodic surface structures	170 fs	Novel single-step and chemical-free technique for ARSS	>99% 8%	0.5–0.7

Note: *T*_d_ means transmittance with double-layer ARSS; *E*_ff_ is used to evaluate the antireflective effect via *E*_ff_ = *T*_d_/*T*_0_ – 1; and *T*_0_ is the bare sample without ARSS.

## 4. Application of ARSSs

As mentioned, the performance of coating technology is dependent on the incident wavelength and angle to a great extent [16]. ARSSs have significant broadband and wide-angle stability, making them more suitable for practical applications [97,98]. In addition, some ARSSs are hydrophobic and resistant to friction [13]. This means that they can still function properly even in adverse weather conditions like rain, fog and wind, reducing the impact of water droplets or dust on the surface [6]. Figure 13 demonstrates the applications of ARSSs in these fields.

For display devices, sunlight readability is a critical requirement. In 2017, Tan et al. demonstrated an ARSS surface on a flexible substrate, intended for flexible display applications [99]. They fabricated the ARSS surface with a reflectance <0.23%, as well as robust mechanical characteristics, a small bending radius (8 mm) and hydrophobicity (with a contact angle >100°). This ARSS surface with good hardness, high flexibility and self-cleaning properties is expected to be applied in curved display devices. Due to the existence of Fresnel reflection between air and glass, the energy conversion efficiency of solar cells is limited. ARSSs can enhance the transmittance within the visible range, thereby improving the working performance. In 2019, Luo et al. fabricated an ARSS on glass through an inductively coupled plasma etching process [100]. This glass with the ARSS exhibits excellent antireflective performance, with a reflectance less than 3% in the wavelength range from 570 to 950 nm. Compared to flat glass, when the incident angle increases from 0° to 60°, the energy conversion efficiency of the photovoltaic (PV) module with ARSS glass increases from 4.6% to 9.9% (Figure 13b). In addition, this glass also possesses self-cleaning abilities and an anti-fogging property, which is beneficial for the outdoor operation of solar cells. It can also be applied in flat panel displays, optical lenses and so on. Infrared detection plays a significant role in military fields. Studying infrared window materials is, therefore, of great worth. In 2022, Wang et al. designed and optimized an ARSS on a ZnS substrate, fabricating using a femtosecond laser Bessel beam [70] (Figure 13c). In their experiment, the ARSS with the optimal geometry expresses high transmittance in the wavelength of 8–14 μm. What’s more, the contact angle of the ARSS reaches 170° for a scanning speed of 9mm/s. With the ARSS, thermograms measured in the infrared detection system are clearer as the temperature increases.

## 5. Conclusions and Outlook

In conclusion, ARSS surfaces based on micro/nanostructures exhibit outstanding optical performance of broadband transmittance, omni-directionality and polarization insensitivity. ARSS surfaces could be scaled up to large areas to enhance the efficiency of solar cells, light emitting diodes as well as optoelectronic and electronic–optical devices like lenses, display screens and photodetectors [95]. In this review, the mechanisms of two antireflection methods, antireflection coatings and ARSSs, have been briefly introduced. Several methods for the preparation of ARSSs, as well as their superiorities and drawbacks, have been discussed. As a flexible and versatile tool, the femtosecond laser has proved to be a micro/nanostructure fabrication method that can be utilized on almost any material. Some simple methods and research progress on fabricating ARSSs using femtosecond laser have been summarized. The surface quality has been enhanced through the use of advanced etching techniques and sacrificial layers as auxiliary support for femtosecond laser processing. However, there are still some challenges that need to be overcome. Using a high-power laser for ablation can result in low precision and high surface roughness [45]. This point-by-point direct-writing method has limited practical applications due to its low efficiency. Currently, ARSS surfaces are mostly limited to flat surfaces, while there is a significant demand for optical windows with curved surfaces. How to realize the fabrication on curved surfaces is an urgent research matter. The ARSS surfaces working in harsh environments require properties such as mechanical stability, thermal stability, super-hydrophobicity and resistance to friction. It is crucial to explore methods for the preparation of comprehensive and multifunctional ARSS surfaces. Although there are still some issues with femtosecond laser processing for ARSSs, recent research has proposed methods to address these problems. With the further exploration and development of processing techniques, femtosecond laser processing is expected to have broader application prospects as scientists delve deeper into understanding the interaction mechanism between lasers and materials.

## Data Availability

No new data were created or analyzed in this study. Data sharing is not applicable to this article.
